# Haplotype Analysis of DXS548 and FRAXAC1 Microsatellite Loci in Iranian Patients with Fragile X Syndrome

**Published:** 2018

**Authors:** Seyed ahmad ALEYASIN, Fatemeh SALAMAT, Mojgan MIRAKHORI

**Affiliations:** 1 Medical Genetic Department, National Institute of Genetic Engineering and Biotechnology, Tehran, Iran.

**Keywords:** FXS, DXS548, FRAXAC1, Haplotype

## Abstract

**Objective:**

Fragile X syndrome (FXS) is the most common cause of inherited mental retardation caused by expansion of a (CGG) repeat region up to 1000 repeat in 5' region of the FMR1 gene located in FRAXA locus Xq27.3. To better understand the mechanism involved in expansion of CGG region, the molecular characteristic of the flanking microsatellite markers in the region must be clarify in different populations. We aimed to examine the potential association between specific haplotype and the expanded AC-repeat region in cases and controls chromosomes.

**Materials & Methods:**

Forty unrelated FXS males and 62 unrelated normal males originating from various regions of Iran were haplotyped by analyzing two CA-repeat markers, FRAXAC1 and DXS548.

**Results:**

Significant linkage disequilibrium was obtained between DXS548 and FRAXAC1 specific marker alleles and CGG repeat expansion among 40 fragile X cases compared to 62 normal controls. The frequencies of DXS548 and FRAXAC1 longer alleles in patients were significantly higher than that in control group. Two FRAXAC1 long alleles were only observed in cases, possibly due to concatenated mutations. The increase of heterozygosities in fragile X cases (DXS548 78.6%, FRAXAC1 64.6%) in comparison to the controls (DXS548 63.0%, FRAXAC1 47.0%) showed a multimodal distribution of fragile X associated alleles.

**Conclusion:**

Haplotype analyses with DXS548 and FRAXAC1 markers represented that haplotype distribution in the normal controls and FXS patients were significantly different, representing a weak founder effect.

## Introduction

Fragile X syndrome (FXS) is the most common cause of inherited mental retardation observed approximately 1 in 4000 males and 1 in 8000 females (1, 2). Males with FXS usually have mental retardation and often exhibit characteristic physical features such as macroorchidism and distinct facial features including long face, large ears, and prominent jaw. Affected females exhibit a similar but usually less severe phenotype. It is caused by the unstable expansion of a CGG repeat within the FMR1 gene and abnormal methylation that induced decreased level of FMR1 mRNA and its FRMP level in the brain (3). The diagnosis of FXS was originally based on the expression of a folate-sensitive fragile site at Xq27.3 (FRAXA) induced in cell culture under conditions of folate deprivation in cytogeneic test. Interpretation for fragile site on X chromosome in cytogenetic test has low resolution and complicating by the presence three other fragile sites, 1 proximal and 2 distal to *FRAXA*, termed *FRAXD*, *FRAXE* and *FRAXF* respectively (4, 5). 

FMR1 gene is located in FRAXA locus contained a tandemly repeated trinucleotide sequence (CGG) near its 5' end. The mutation responsible for FXS involves the expansion of this repeat segment. The number of CGG repeats in the FMR1 gene of the normal population varies from 6 to approximately 50. There are two main categories of mutations, premutations of approximately 60 to 250 repeats and full mutations of more than approximately 250 repeats. There is no clear boundary between the upper limit of normal and the lower limit of the premutation range. For this reason alleles with approximately 45-55 copies of the repeat are said to be in the "gray zone". Some alleles in this size range are unstable and expand from generation to generation, while others are stably inherited. A premutation is susceptible to expansion after passage through a female meiosis. The larger size of a mother's premutation the more the risk of expansion to a full mutation in her offspring (6).

Transporter of a premutation is not FX. Male transporters are nominated as "normal transmitting" males and they transfer the mutation to their daughters without a length change. These daughters are unaffected, but are at risk of having affected offspring. Variable clinical severity is observed in both sexes. Most, but not all, males with a full mutation are mentally retarded and show typical physical and behavioral features. Of females with a full mutation, approximately one-third is of normal intelligence, one-third is of borderline intelligence, and one-third is mentally retarded. 

Inheritance of FX mutations, are more complicated than common classic Mendelian traits. While passage through a female meiosis is necessary for significant trinucleotide repeat expansion, the most likely expansion occurs during early embryonic development. Since expansion occurs in a multicellular embryo and the extent of expansion may vary from cell to cell, individuals often display somatic heterogeneity in allele size. Some affected individuals, termed mosaics, exhibit both a premutation and a full mutation in blood. 

A full mutation contained more than 200 CGG repeats is commonly associated with gene promoter methylation and is related to gene inactivation. Gene inactivation is main cause of FX phenotype. However, its impact on severity of its clinical presentation is somehow uncertain mainly in females (7). 

Two microsatellite AC-repeat markers, DXS548 and FRAXAC1, have demonstrated significant linkage disequilibrium at the FMR1 locus in populations from various geographic areas. They located 150 kb and 7 kb centromeric proximal to CGG repeats, respectively, and known the most widely used markers for truncated STR-based haplotyping in investigations on FMR1 and its correlations with FXS (8). The utility of microsatellite markers for association studies has reduced with the developments of direct testing it has been recommended for laboratories to have access to this marker for occasional unusual or complicated cases especially when mother of cases is carrier for CGG repeat expansion and already had an affected son. Using these markers the segregation of affected chromosome can be follow up in suspected cases even in a multiplex PCR. 

This is the first study has aimed to detect the correlation between CGG repeat haplotype of the two polymorphic markers DXS548 and FRAXAC1 and Iranian FXS compared to the distribution of those haplotypes in normal Iranian population in who living in and around Tehran. The expansion of FRAXA related microsatellites could help application of these molecular markers in diagnosis of FXS as well as better understanding of genomic instability in the FRAXA locus in FXS.

## Materials & Methods 

Subjects selected for DXS548-FRAXAC1 haplotype analysis included 40 unrelated fragile-X male patients and 62 unrelated normal males. DNA of patients was donated by Dr H. Najmabadi, University of Social Welfare and Rehabilitation Sciences, Tehran, Iran, Tehran, Iran. All patients had been identified to have fragile site at Xq27.3 (FRAXA) using PCR and Southern blot analysis. Controls were 62 age/sex matched unrelated healthy subjects from the same geographic area. Genomic DNA was extracted from peripheral blood leukocytes using a commercial kit (QIAamp DNA Mini Kit). 

Informed consent was obtained from all participants and the study was approved by the Ethics Committee of the center. 

**CGG Repeat Analysis**

To describe the general distribution of the CGG repeat structures within an unaffected Iranian population, the numbers of FMR1 CGG repeats were determined initially in 62 chromosomes of Iranians living in Tehran City that were from different cities of Iran related to various indigenous communities. PCR analysis for detection of CGG repeat in the FMR1 gene was performed by PCR-PAGE method using forward ^5^^'^agccccgcacttccaccaccagctcctcca^3^^' ^and reverse ^5^^'^cgacctgtcaccgcccttcagccttcc^3^^' ^primers (9). PCR conditions was started by an initial denaturation at 95 ˚C for 5 minute, followed by 10 cycles of 95 ˚C for 1 min, 65 ˚C for 1.5 min, and 72 ˚C for 1.5 min and 30 cycles with 55 ˚C for 1.0 min annealing temperature. Each 20 µl reaction contained 2.0 µl Taq buffer, 0.5 µM each primers, 200 µM each dNTPs except for 50 µM dGtp and 150 µM 7deza dGtp, 5% DMSO and 1unite *Pfu* polymerase. The products were resolved on 8% denaturing polyacrylamide gels and visualized by silver staining. A control sample of about 50 repeats was always considered for comparison.

**DXS548-FRAXAC1 Haplotype**

The DXS548 AC-repeat polymorphism was amplified using forward ^5^^'^gtacattagagtcacctgtggtgc^3^^'^ and reverse ^5^^'^agagcttcactatgcaatggaatc^3^^' ^primers in amplification conditions described by Riggins et al (10). FRAXAC1 polymorphism was amplified using forward ^5^^'^gatctaatcaacatctatagactttatt^3^^'^ and reveres ^5^^'^gatgagagtcacttgaagctgg^3^^'^ primers (11μμΜμ_2_, and 0.5U Taq DNA polymerase (Roche, Germany). PCR thermal cycle was performed in 32 cycles. Each cycle consisted of 95 °C denaturizing for 30 seconds, 60 °C annealing for 1 minute and 72 °C extension for 40 seconds. The thermal cycles were started with an initial denaturizing of 95 °C for 5 min and a final of 72 °C extension for 10 min.

Evaluation of polymorphism was carried out using denaturing polyacrylamide gel electrophoresis contained 8% polyacrylamide (29:1 acrylamide/bisacrylamide), 7M urea, 1XTBE, 500 μl 10% ammonium persulfate, and 50 μl TEMED. Gels were leaved to polymerize at room temperature for 1 hour. The gel apparatus was DNA sequencing unit (30 X 45 cm) connected to 2000V power supply clamps. PCR products (25 μl) were mixed with 45 μl of loading buffer and denatured at 95 °C for 8 min. Denatured samples were cooled on ice and 8μl of each sample was loaded. Gels were run at 50 μC constant temperature and 100 watts limiting power for about 3 hours (BioRad Power/Pak 3000 power supply). Gels were stained and alleles were visualized using standard silver staining procedure.

**Statistical Analysis**

DXS548 and FRAXAC1 alleles were named by base pair marking (12). For representation of haplotypes, DXS548 alleles have been introduced as reported earlier (11, 13) and for FRAXAC1 alleles were introduced (14, 15).

Expected marker heterozygosity was calculated using the formula $1-{\sum q}^{2}$ where "q" is the frequency of each individual allele at FRAXAC1 and DXS548 locus. The Chi- square test was employed to test significance of differences between allele frequencies and haplotypes in fragile X patients and controls, SPSS 12.0 software (Chicago, IL, USA).

## Results

**CGG Repeat Analysis**

General distribution of the CGG repeat within 62 unaffected Iranian population resulted in variable alleles, ranging in size 11-38 repeats, were detected as alleles 26 (13.0%), 29 (24.0%), and 33 (11.0%) being the most common (Table 1).

**Table 1:** Distribution of (CGG)n Alleles in 62 normal X Chromosomes of Iranian

**CGG Repeat Number**	***N***	***f***
11	1	0.016
12	1	0.016
13	0	0.000
14	1	0.016
15	0	0.000
16	0	0.000
17	0	0.000
18	0	0.000
19	1	0.016
20	0	0.000
21	5	0.080
22	1	0.016
23	1	0.016
24	0	0.000
25	1	0.016
26	8	0.129
27	2	0.032
28	2	0.032
29	15	0.241
30	1	0.016
31	3	0.048
32	2	0.032
33	**7**	0.112
34	1	0.016
35	4	0.065
36	3	0.048
37	1	0.016
38	1	0.016
Total heterozygosity	62	1.0

*N*=number of X chromosomes; *f*=frequency.

**Allele Polymorphism at DXS548 and FRAXAC1**

The distribution of DXS548 and FRAXAC1 alleles in subjects of normal controls and FXS is listed in Table 2. The DXS548 alleles were 9 (190), 8 (192), 7 (194), 6 (196), 5 (198), 4 (200), 3(202), and 2 (204) and the FRAXAC1 alleles were E (104), D (106), C (108), B (110), and A (112) respectively.

In the 62 normal X chromosomes, we observed 6 alleles in DXS548 locus (2, 3, 4, 5, 6, 7, 8 and 9 corresponding to PCR products of 202, 200, 198, 196, 194, 192 and 190bp, respectively) that the most frequent DXS548 allele was allele 7 (58%), followed by allele 6 (14.9%) (Table 2). In FXS group 5, however, alleles 2, 3 ,4 , 6, and 7 were observed that alleles 3 and 6 were the most common alleles (25%). 

**Table 2:** Allele Frequency of DXS548 and FRAXAC1

^bp(ACs)/Allele^	^Control (%)^	^FXS(%)^
^DXS548^		
^190 (18)/9^	^ 1(1.6) ^	^0^
^192 (19)/8^	^3(4.8)^	^0^
^194 (20)/7^	^36(58^^)^	^9(22.5)^
^196 (21)/6^	^9(14.6)^	^10(25)^
^198 (22)/5^	^2(3.22)^	^0(0)^
^200 (23)/4^	^6(4.67)^	^6(15)^
^202 (24)/3^	^3(4.83)^	^10(25)^
^204 (25)/2^	^ 2(3.22) ^	^5(12/5)^
^206 (26)/1^	^0^	^0^
^Total^	^62^	^40^
^Heterozygosity^	^37.06%^	^ 78.63%^
^FRAXAC1^		
^104(17)/E^	^4(6.45)^	^4(10)^
^106(18)/D^	^28(45.1)^	^5(12.5)^
^108(19)/C^	^30(48.3^^)^	^22(55)^
^110(20)/B^	^0 ^	^5(12.5)^
^112(21)/A^	^0^	^4(10)^
^Total^	^40^	^62^
^Heterozygosity^	^53.07%^	^35.37%^

As shown in Table 2 up to five FRAXAC1 alleles including alleles E, D, C, B and A were observed in the fragile X chromosomes and three alleles C , D and E in control group with a predominance in the allele C among both groups (%55 in FXS group and 48.3% in control group). The allele distributions at DXS548 locus and FRAXC1 in the controls and FXS groups were significantly different, as *P*=0.001 and *P*=0.00, respectively. Heterozygosity of DXS548 was 37.06% in controls and increased to 78.68% in the FXS group. Observed heterozygosities for FRAXAC1 were 53.07% and 35.37% in control and FXS groups, respectively.

**Haplotype Analysis**

Distribution of haplotypes in 62 normal individuals with different CGG repeats is shown in Table 3. The CGG repeats >29 was recognized in 22 normal individuals and was related with haplotypes 7D {31, 33, 36, 35 repeats}, 7C {32, 33}, 6C {31}, 4C {30, 31, 33}, 6D {34}, 3C {35, 37}, 5D {36}, 8C {38}, and 9D {35}. Haplotypes 5D, 7E, 2C, 8D, and 4D were only observed in lower number of CGG repeats of normal individuals with <28 repeats.

**Fig.1:**
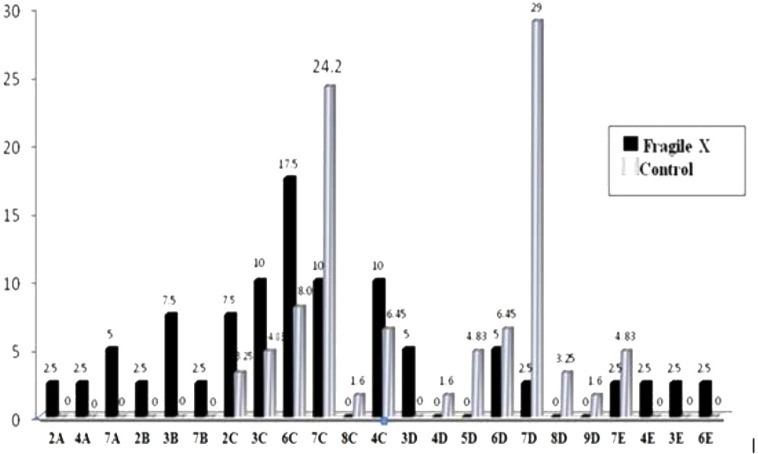
DXS548–FRAXAC1 haplotype frequency distribution in unrelated non-fragile X control chromosomes (n=62) and fragile X chromosomes (n=40) from the Iranian population. Label of each bar indicates the haplotype percentage

Figure 1 shows DXS548-FRAXAC1 haplotype frequency distribution in control (n=62) and FXS (n=40) subjects. Haplotypes 7D (29%) and 6C (17.5%) were presented in higher incidence among control and FXS groups, respectively. The distribution of DXS548 allele size in our Iranian subjects with fragile X syndrome has been compared with published data in other countries in Table 4. 

**Table 3:** Haplotype distribution of DXS548-FRAXAC1 among 62 normal individuals

Haplotype	N (%)	n(CGG repeats)
7D(194-106)	18(29)	1(12), 1(14), 1(19), 4(26), 1(27), 1(28), 1(29), 1(31), 3(33), 2(35), 2(36)
7C(194-108)	15(24.2)	3(21), 1(25), 7(29), 2(32), 2(33)
6C(196-108)	5(8.06)	2(26), 2(29), 1(31)
4C(200-108)	4(6.45)	1(23), 1(30), 1(31), 1(33)
6D(196-106)	4(6.45)	1(28), 2(29),1(34)
3C(202-108)	3(4.83)	1(23),1(35), 1(37)
5D(198-106)	3(4.83)	2(29), 1(36)
7E(194-104)	3(4.83)	1(11), 1(22), 1(29)
2C(204-108)	2(3.25)	1(26), 1(29)
8D(192-106)	2(3.25)	1(21), 1(29)
8C(192-108)	1(1.6)	1(38)
4D(200-106)	1(1.6)	1(29)
9D(190-106)	1(1.6)	1(35)
Total haplotype13	62	62

n=number of patients

**Table 4:** DXS548 allele size distribution in subjects with fragile X syndromes in Iran compared published data

DXS548	206	204	202	200	198	196	194	192	190	other	Total	Reference
	1	2	3	4	5	6	7	8	9			
Iran	0	5	10	6	0	10	9	0	0	0	40	Present study
USA	2	21	11	6	1	26	49	9	0	0	125	Oberle 1991
France & Spain	1	29	1	1	0	32	42	0	0	0	106	Smits 1993
Belgium-Holand	0	25	0	1	0	14	27	1	0	0	68	Richards 1992
N Eroup& USA	3	13	3	2	1	6	22	0	0	0	50	Zhong 1994
Italy	7	36	12	2	0	24	36	7	0	1	120	Oudet 1993
UK	1	8	0	0	0	16	15	4	0	0	44	Buyle 1993
Sweden	0	2	0	0	0	12	14	0	0	0	28	Riggings 1992
Finland	0	5	0	0	0	0	54	1	0	0	60	Chiurazzi 1994
Finland	0	2	0	0	0	0	35	0	0	0	37	Macpherson 1994
Finland	0	2	0	0	0	0	24	0	0	0	26	Malmgren 1994
Portugal	0	15	0	0	8	9	9	0	0	1	42	Peixoto 1998
Spain	0	10	0	0	1	3	5	0	0	0	19	Yolando 2002
Indian	0	1	0	0	0	0	6	9	5	0	21	Chakraborty 2008
Taiwan	0	0	0	0	0	0	27	1	0	0	28	Tzeng 2005
Central mainland of China	0	0	0	0	0	20	6	1	0	0	27	Zhong 1994 &1999

## Discussion

We detected 21 different normal alleles ranging in size from 11 to 38 CGG repeats as alleles 26 (13.0%), 29 (24.0%) and 33 (11.0%) being the most common. The most common fragile X CGG repeat was 29 copies in our study. These data are in agreement with findings previously reported in Asian population (16) such as Indian (17) and Andalusia (18).

The increase of heterozygosities has been observed in FXS samples for DXS548 (78.63%) locus relative to the controls (37.07%). The increase of heterozygosity in FXS patients have been observed in nearly every population studied (12, 19-21) and this indicants multimodal distribution of the fragile X linked alleles and the presence of one dominant allele in the controls. The most frequent allele in our control group was allele 7 (194 bp) observed in 58% of individuals. A unimodal distribution with a high frequency of 80–90% of the 194 allele is also recognized in many populations like the Caucasians, Chinese, Japanese, and the Thais. In control group, the modal allele 194 was flanked by 196 at a frequency of 14.6%. However, the 194 and 192 alleles were present in approximately equal frequencies 43% and 41%, respectively, in two different ethically population of eastern and north India (22,23) while the frequency of 192 allele (4.8%) was not as high as frequency of allele 194 in the present study. We detected the 204 allele at a lower frequency (3.22%) in agreement with the common origin of the Indo-European populations. Table 4 summarized the frequencies of alleles of DXS548 locus in FXS subjects in the major populations of the world and the studied population. Comparison of the frequency of alleles between FXS and control subjects reveals a significant *P* value <0.001 and chi square 23.66 representing association between longer allele in DXS548 and FXS due to either founder effect or concatenated mutation. The heterozygosity of our normal group (37.06%) is higher than the Chinese (33%) and Thai (16.5%) population which indicate the heterogeneity of the studied Iranian population by us.

Moreover the number of longer alleles was higher in FXS samples than that of controls for both tested microsatellite markers. The percentage of longer alleles 2 and 3 for DXS548 in cases group was 12.5% and 25% in comparison to 3.2% and 4.8% in control group respectively. The percentage of longer alleles A and B for FRAXAC1 in FXS group was 10% and 12.5% in comparison to 0.0% in control group. In addition, some short alleles such as 8 and 9 for DXS548 were only observed in control group equal to 6.5%. FRAXAC1 alleles distribution showed five different kinds of allele in our cases (A, B, C, D, and E) and three (C, D, and E) in normal population. The alleles A (21 repeat) and B (20 repeat) were absent in our normal population whereas they were participated in 22.5% of haplotypes in FXS group. Therefore A and B alleles were associated with fragile X chromosomes in our population. The FRAXAC1 locus alleles C (48.3%) and D (45.1%) were modal with a higher frequency in controls, whereas in FXS locus allele C (55%) was modal with a higher frequency in case group. The D and C alleles are the most frequent in Asian and Caucasians populations, respectively (24). Heterozygosity of 53% was comparable to the British (50%) and Chinese (49%).

The DXS548-2 (204bp) allele is known to be common on *FMR1 *founder chromosomes in Europeans (14, 16, 25), but this allele is not observed in Asian fragile X chromosomes (19, 26), while, we observed this allele in FXS group with 12/5%. The individuals included in our study are from Tehran which is a metropolitan city where people from different area of Iran are living. The genetic status of these individuals is thus a complex from different region of Iran and it is not surprising that the allele frequencies observed in the 2 polymorphic loci DXS548 and FRAXAC1 were as a model for Iranian population. The results of the CA repeat analysis at DXS548-FRAXAC1 in the normal X chromosomes and Fragile X chromosomes was 13 and 18 different haplotypes, respectively. There was two single major haplotype within each group as has been found in different ethnic groups of the world (13-15, 19, 23, 25, 28-30) where founder effects have been demonstrated. Of the 18 haplotypes represented in the FXS group, ten haplotypes were not observed in the control group. This suggests the likely of the admixture of immigrant haplotypes in our population, which is highly probable.

The most significant haplotype was 7D represented in 29.0% of the X chromosomes followed by 7C (24.2%) and 6C (8.06%). The modal haplotype 7D, instead of the nearly worldwide haplotype 7C, had been also observed exclusively in Eastern Asian populations (8). Therefore, this haplotype can be considered a hallmark feature distinguishing the Iranian population from the extensive predominance of haplotype 7C observed in normal X chromosomes from most other populations. The modal haplotype 7D in our population was similar with previous report in normal Mexican Mestizo and Indigenous populations (8).

The haplotype 8D, that was not observed in our population, has been exclusively reported almost in East Asian populations (Chinese and Taiwanese) with a significantly lower frequency (1-4%) and the Mexican population (23.8%). Moreover, haplotype 6D detected in 5.3% of the Mexican population is found in most other populations at a comparable frequency (1-13%; except 25% in Eskimos). The second modal haplotype 8D was observed in 23.8% of the Mexican population has been reported almost exclusively in East Asian populations (Chinese and Taiwanese) but at a significantly lower frequency (1-4%). Moreover, haplotype 6D detected in 6.45% of the studied population is reported in most other populations at a comparable frequency (1-13%; except 25% in Eskimos). The haplotype 6C which is 8.3% among normal populations was found to be the most frequent haplotype with frequency of 17.5% in our fragile X population that followed by 3C, 4C and 7C (10%) each. The 6D haplotype has been found to be the most common haplotype among the fragile X chromosome in white Americans, Chinese, and British population. However, it has not been found among the fragile X chromosomes in ours and the Indian, Thai population (23). It occurs in our FXS group at 5% frequency. In addition, 7D is known the most frequent haplotype in Thai population in both controls and cases but 7D was not observed in our population.

Of the 23 haplotypes in FXS group, 10 were not present in the control group. Therefore, these haplotypes were found to be associated with expansion of CGG repeats in our population. This suggests the likely of the admixture of immigrant haplotypes in Iranian population which is highly probable. Despite the lack of single modal haplotype in this highly heterogeneous population, haplotype distribution between the controls and FXS groups is significantly different (P<0.01). This suggests weak founder effect.

**In conclusion, **the CA repeat analysis at DXS548-FRAXAC1 loci in the normal X chromosomes and Fragile X chromosomes represented 13 and 18 different haplotypes, respectively. A few unique longer alleles A and B for FRAXAC1 was found in FXS chromosomes compared to normal controls which represent a weak founder effect.
